# Peripheral inflammation as a potential mechanism and preventive strategy for perioperative neurocognitive disorder under general anesthesia and surgery

**DOI:** 10.3389/fncel.2024.1365448

**Published:** 2024-07-03

**Authors:** Yuan Li, Ying-Jie Li, Xu Fang, Dong-Qin Chen, Wan-Qiu Yu, Zhao-Qiong Zhu

**Affiliations:** ^1^Department of Anesthesiology, Affiliated Hospital of Zunyi Medical University, Zunyi, China; ^2^Department of Anesthesiology, Mianyang Hospital of Traditional Chinese Medicine, Mianyang, China; ^3^Department of General Surgery, Mianyang Hospital of Traditional Chinese Medicine, Mianyang, China; ^4^Department of Anesthesiology, Nanchong Central Hospital, The Second Clinical Medical School of North Sichuan Medical College, Zunyi, China; ^5^Early Clinical Research Ward of Affiliated Hospital of Zunyi Medical University, Zunyi, China

**Keywords:** peripheral inflammation, perioperative neurocognitive disorder, general anesthesia, central nervous system, preventive strategies

## Abstract

General anesthesia, as a commonly used medical intervention, has been widely applied during surgical procedures to ensure rapid loss of consciousness and pain relief for patients. However, recent research suggests that general anesthesia may be associated with the occurrence of perioperative neurocognitive disorder (PND). PND is characterized by a decline in cognitive function after surgery, including impairments in attention, memory, learning, and executive functions. With the increasing trend of population aging, the burden of PND on patients and society’s health and economy is becoming more evident. Currently, the clinical consensus tends to believe that peripheral inflammation is involved in the pathogenesis of PND, providing strong support for further investigating the mechanisms and prevention of PND.

## 1 Introduction

Perioperative neurocognitive disorder (PND) is a well-known central nervous system (CNS) complication, particularly prevalent in older individuals, associated with alterations in cognitive function following general anesthesia (Bilotta et al., 2016; Evered and Silbert, 2018). These changes encompass impairments in attention, memory, learning, and executive functions, among others. A comprehensive review of clinical trials and observational studies worldwide revealed varying incidences of PND following general anesthesia and surgery, ranging from 10 to 70% (Peters van Ton et al., 2021). These discrepancies may stem from differences in research methodologies, criteria for defining PND, and patient characteristics (Evered et al., 2011; Paredes et al., 2016; Klinger et al., 2018). High-risk patients, such as the elderly, those with pre-existing cognitive impairments, and those who experience complications during surgery, are more susceptible to cognitive decline after general anesthesia (Price et al., 2008; Lin et al., 2012). Moreover, the type of general anesthesia may also play a role in the occurrence of PND. Some studies have indicated a higher risk of PND with inhalation anesthesia, while intravenous anesthesia may be associated with a lower risk (Tang et al., 2014). The incidence rates of PND following various types of surgeries under general anesthesia also exhibit significant variations. A prospective multicenter study conducted by International Study of PND in 1998 revealed that among 1,218 elderly patients undergoing non-cardiac surgeries under general anesthesia, the incidence rates of PND at 1 week and 3 months postoperatively were 26 and 10%, respectively (Moller et al., 1998). Research by Newman et al. indicated that in 261 patients undergoing coronary artery bypass grafting, the incidence rates of PND at discharge, 6 weeks, 6 months, and 5 years postoperatively were 53, 26, 24, and 42%, respectively (Newman et al., 2001). A study by Koch et al. showed that in 24 patients undergoing knee or hip replacement surgeries, the incidence rates of PND at discharge and 3 months post-discharge were 75 and 45%, respectively (Koch et al., 2007). Other reports suggest that the incidence rate of PND following cardiac surgeries can reach as high as 60%, significantly higher than that of other surgeries, which may be attributed to factors such as the extensive trauma and susceptibility to infections associated with cardiac surgeries (Polunina et al., 2014).

To gain a deeper understanding of the impact of general anesthesia on cognitive function, many studies have focused on the role of peripheral inflammation in influencing brain structure and function. Peripheral inflammation is an inflammatory response that occurs in the peripheral tissues of the body, and this response can affect cognitive function through neuro-immune pathways (Wilson et al., 2002). Research indicates that peripheral inflammatory response can lead to the release of inflammatory factors, which can influence cognitive function and behavioral performance through neural pathways. To be more specific, the inflammatory factors are able to breach the blood–brain barrier (BBB), disrupting the normal activity of neurons and consequently leading to cognitive impairments (Sheeran and Hall, 1997; Quan et al., 2019). In this comprehensive review, we first introduce the peripheral inflammation and its underlying mechanisms. Subsequently, we delve into the relationship between peripheral inflammation and the CNS as well as PND. Then, we summarize the regulation of general anesthetics and auxiliary drugs on peripheral inflammation. Finally, we summarize relevant prevention strategies, along with the latest research developments at the cellular and molecular levels. The aim of this paper is to provide a more insightful understanding of the occurrence, development, and preventive strategies for PND, offering important references and insights for clinical practice and future research to aid in the management of these vulnerable elderly patients.

## 2 Methods

Two investigators (YL and Y-JL) conducted a systematic search of three electronic databases (MEDLINE via PubMed, Embase via Ovid, and Web of Science) to identify studies published until December 2023. The search strategy included the terms: “(“inflammatory” OR “inflammatory response” OR “cytokine”) AND (“perioperative neurocognitive disorder” OR “postoperative cognitive dysfunction” OR “postoperative cognitive impairment” OR “postoperative cognitive decline” OR “postoperative cognitive disorder”) AND (“general anesthesia” OR “anesthesia” OR “surgery” OR “operation”).” Studies investigating the association between inflammatory markers and perioperative neurocognitive disorder were included. Only articles written in English were considered. Exclusion criteria comprised case reports, editorials, correspondences, and clinical guidelines. Subsequently, two investigators independently screened the titles and abstracts of all studies based on the eligibility criteria. Any relevant articles identified by either investigator underwent full-text review.

## 3 Peripheral inflammation and its mechanisms

Peripheral inflammation refers to the inflammatory response that occurs in various tissues and organs outside the CNS. These tissues and organs include but are not limited to the skin, muscles, joints, lungs, kidneys, and heart. Peripheral inflammation can be caused by various factors, including infection, injury, and autoimmune reactions. In peripheral tissues, the occurrence of inflammatory reactions is a defense mechanism of the immune system against external stimuli or abnormal conditions (Xu and Larbi, 2018). Different peripheral tissues may exhibit different manifestations and degrees of inflammatory reactions. For example, the inflammatory reaction in the skin often manifests as redness, swelling, and pain, while the inflammatory reaction in muscles and joints can cause muscle pain and joint swelling, peripheral inflammation in the lungs may manifest as symptoms such as cough, difficulty breathing, and lung infections, while inflammation in the kidneys and heart can cause damage and functional abnormalities in the respective organs (Beltrani and Beltrani, 1997; Lotz and Kraus, 2010; Scott et al., 2012; Peerapornratana et al., 2019; L'Heureux et al., 2020). Peripheral inflammation has a significant impact on overall health. The inflammatory response helps clear pathogens, repair tissue damage, and promote the coordinated action of immune cells (Kiecolt-Glaser et al., 2002). However, excessive or prolonged peripheral inflammation can lead to the development of diseases such as inflammatory diseases, autoimmune diseases, and neurological disorders (Pascoal et al., 2022). Research has shown that in the onset and progression of Alzheimer’s disease (AD), chronic inflammation can lead to damage and apoptosis of neurons. Excessive production of inflammatory mediators may trigger neuroinflammatory responses, resulting in structural and functional impairment of neurons, affecting the normal communication within neuronal networks (Paouri and Georgopoulos, 2019). The inflammatory state also impacts the metabolism and clearance of amyloid proteins, leading to increased deposition in the brain (Xie et al., 2021). Interleukin-1beta (IL-1β) and interleukin-6 (IL-6) can influence β-amyloid precursor protein and promote its production in an inflammatory environment (Del Bo et al., 1995). Tumor necrosis factor- alpha (TNF-α) can accelerate the aggregation of amyloid proteins and exert toxic effects on neurons (Decourt et al., 2017). Furthermore, studies indicate that long-term chronic inflammation and excessive production of inflammatory mediators may trigger inflammatory processes, activating microglial cells in the brain, leading to dopaminergic neuronal degeneration and increasing the risk of Parkinson’s disease (Marogianni et al., 2020). Recent research has also found that patients with schizophrenia often exhibit high levels of peripheral inflammatory markers, such as C-reactive protein (CRP) and pro-inflammatory factors (Müller, 2018; Murphy et al., 2022). Changes in the expression of regulatory nuclear factor κB (NF-κB) pathway mRNA associated with peripheral inflammation may be related to cognitive impairment in schizophrenia and more severe psychiatric symptoms (Murphy et al., 2022). The generation of peripheral inflammation involves multiple mechanisms.

Activation of immune cells: When the body is exposed to stimuli, various types of immune cells in the immune system are activated, participating in the modulation of immune and inflammatory responses. These cells include monocytes (such as macrophages), T cells, B cells, and natural killer (NK) cells, among others. Macrophages are important members of the immune system that can engulf and digest pathogens, cellular debris, and other abnormal cells (Wynn and Vannella, 2016). When macrophages are stimulated, they release various cytokines such as TNF-α, IL-1β, and interleukin-6 (IL-6), which play important regulatory roles in inflammatory reactions. They can induce the activation and migration of other immune cells, promoting the occurrence and development of inflammation (Bruscia and Bonfield, 2016; Decano et al., 2016). T cells and B cells are major cell types in the immune system that play crucial roles in immune responses. When stimulated, T cells and B cells regulate and participate in immune responses by releasing cytokines and antibodies, respectively. T cells can differentiate into different subtypes, such as helper T cells and cytotoxic T cells, which regulate and mediate immune responses, respectively (Wang et al., 2023). B cells can differentiate into plasma cells that produce antibodies to neutralize pathogens and participate in immune responses (Nutt et al., 2015). NK cells are a type of lymphocytes with cytotoxicity that play important roles in early immune responses, they can recognize and kill infected or abnormal cells, releasing cytokines and mediating inflammatory responses (Abel et al., 2018). Upon sensing stimulus signals, these immune cells modulate and activate the functions of other immune cells by releasing cytokines and other regulatory molecules, thereby participating in immune responses and inflammatory reactions (Dirchwolf et al., 2016). Their activation and interactions are important mechanisms for maintaining immune system balance and protecting overall health.

Release of inflammatory mediators: Inflammatory mediators are a class of molecules that play crucial roles in inflammatory reactions. Inflammatory mediators can be produced by various cell types in cells, tissues, or organs, including immune cells, endothelial cells, and fibroblasts, among others (Yuan et al., 2016). These mediators can activate, recruit, and regulate immune cell functions, triggering inflammatory reactions (Iwasaki and Medzhitov, 2010). Cytokines are among the most important mediators in inflammatory reactions. TNF-α, IL-1β, and IL-6 are a few common cytokines. When the immune system is stimulated by certain triggers, such as infection, injury, or underlying medical conditions, immune cells are activated and release these mediators. These mediators act on target tissues, causing local inflammatory reactions (Maclullich et al., 2008). The release of cytokines also attracts other immune cells to the site of inflammation, contributing to the amplification and maintenance of the inflammatory response (Peck and Mellins, 2010). Chemokines are another class of mediators in inflammatory reactions, they can attract immune cells to the site of inflammation, among the most prevalent chemotactic factors are CC chemokines and CXC chemokines (Le et al., 2004). CC chemokines are likely to act on monocytes and lymphocytes whereas CXC chemokines tend to attract neutrophils and lymphocytes to inflammatory sites (Wallace et al., 2004). Moreover, pro-inflammatory cytokines are also important mediators that play significant roles in inflammatory responses, they not only activate immune cells but also promote tissue cell repair and recovery after inflammation (Chanana et al., 2006). Transforming growth factor-beta-inducing protein-1 and fibroblast growth factors are common pro-inflammatory cytokines. In summary, inflammatory mediators are indispensable components of inflammatory reactions. By releasing these mediators, immune cells can engage in essential processes in various inflammatory reactions, including recruitment of immune cells, effects of cytokines, expansion of inflammation, and repair.

Unfolding of inflammatory responses: The unfolding of inflammatory responses is a complex process involving interactions among immune cells, inflammatory mediators, and related tissues and organs. When the body’s tissue is damaged, immune cells are triggered and begin to release inflammatory mediators, such as cytokines and chemokines, these mediators can cause a series of biological reactions at the site of inflammation, leading to the unfolding of the inflammatory response (Ramirez et al., 2018). Among these reactions, vasodilation and increased vascular permeability represent the earliest stages of the inflammatory response. These changes result in increased local tissue blood flow and leakage of fluid from blood vessels, leading to significant increases in local temperature and swelling (Pober and Sessa, 2014). Vasodilation at the site of inflammation facilitates increased local blood flow to bring immune cells, drugs, and other biologically active molecules to eliminate damage and pathogens, thereby promoting the resolution of inflammation (Colling et al., 2021). The infiltration of cells and fluid is another important characteristic of the inflammatory response. During inflammation, immune cells are activated and migrate to the site of inflammation, causing the infiltration of various immune cells, such as lymphocytes, macrophages, and neutrophils, within a short period of time, further enhancing the unfolding of the inflammatory response (Coussens and Werb, 2002). In addition, the leakage of fluid at the site of inflammation can also cause tissue swelling, intensifying local pathological reactions (Akiyama et al., 2022). The unfolding of the inflammatory response is a complex and dynamic process involving interactions among various cells and molecules. The occurrence and development of inflammation are closely related to the mechanisms of the body’s immune response, which ensures the rapid and effective response to trauma, infection, or other external stimuli. However, excessive or prolonged inflammatory responses can lead to a range of inflammatory diseases.

## 4 Relationship among peripheral inflammation, central nervous system, and perioperative neurocognitive disorder

**Figure 1:**
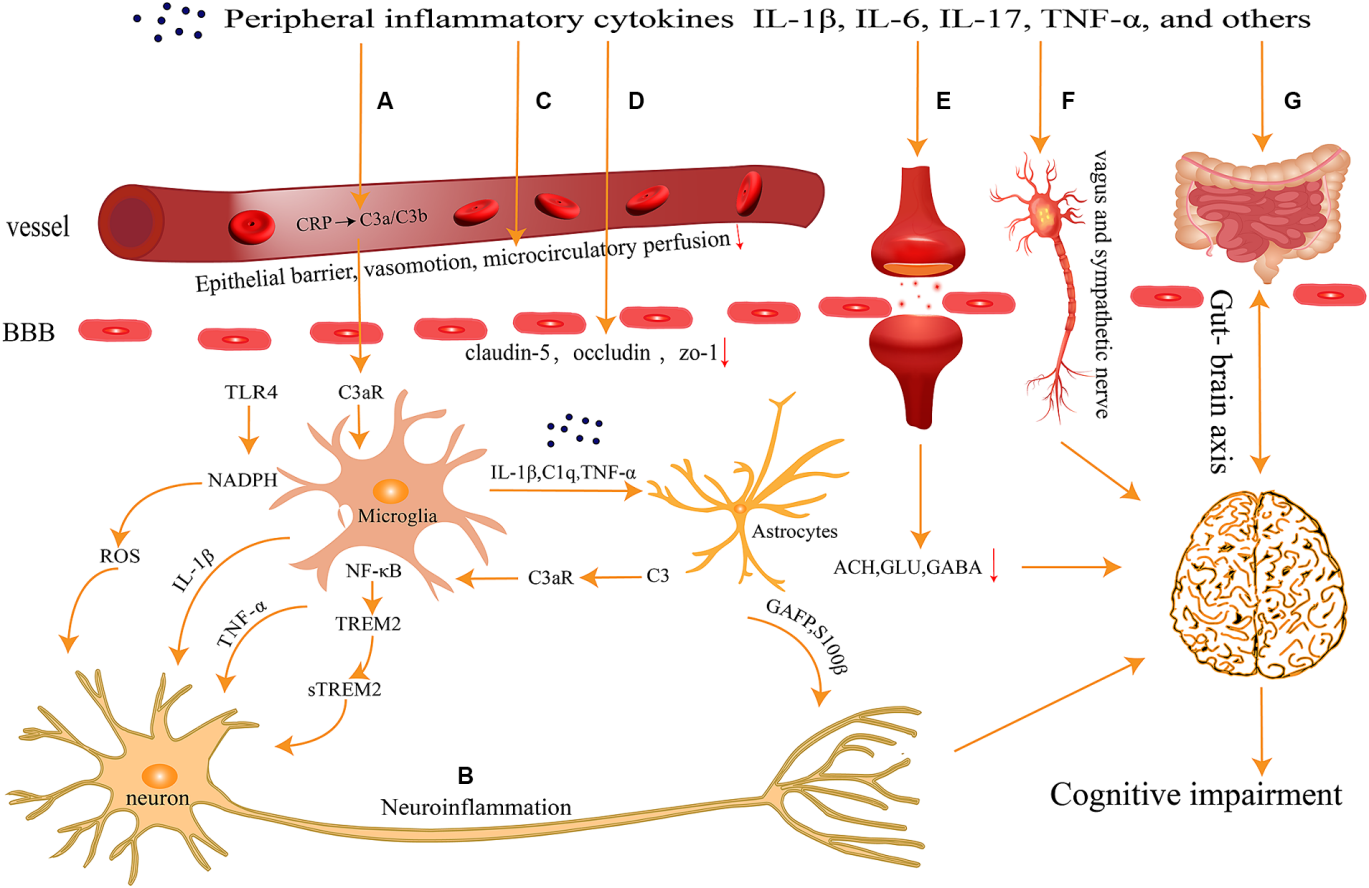
Peripheral inflammation can spread to the central nervous system through several pathways **(A)** activation of the complement system; **(B)** neuronal inflammatory response; **(C)** abnormalities in vascular function and hemorheology; **(D)** breakdown of the Blood–Brain Barrier; **(E)** imbalance of neurotransmitters; **(F)** neural transmission pathways; and **(G)** gut-brain axis, ultimately contributing to cognitive impairments.

The CNS has long been regarded as the “forbidden zone” of the body because of the presence of the BBB and the blood-cerebrospinal fluid barrier, which render the brain immune-independent and unaffected by the external immune system. However, recent research by Iliff and colleagues has revealed the existence of lymphatic pathways in the CNS (Iliff et al., 2015), while another research group has discovered that the meningeal lymphatic pathway in the CNS is interconnected with the peripheral immune system (Louveau et al., 2015). These findings provide new clues for a better understanding of the interactions between the CNS and the immune system, overturning the traditional assumption of the brain as an immune-privileged organ. Peripheral inflammation response leads to increase in levels of inflammatory factors such as IL-1β, IL-6, TNF-α in plasma, which diffuse through various pathways into the CNS and ultimately result in cognitive impairment (Figure 1).

### 4.1 Breakdown of the blood–brain barrier

BBB is a complex CNS structure that precisely regulates the transport of ions, molecules, and cells between the CNS and the peripheral system (Daneman, 2012). The BBB plays a protective role in preventing brain damage and maintaining a normal biochemical microenvironment. However, in a state of peripheral inflammation, inflammatory mediators may cross the BBB and directly affect neuronal function (Tonelli, 2015). These inflammatory mediators, such as TNF-α, IL-1β, and IL-6, can enter the brain through various pathways and interfere with normal neuronal function (Mohagheghi et al., 2013). The persistent presence of these inflammatory mediators may lead to the sustained activation of neuronal inflammation and have detrimental effects on cognitive function.

Peripheral inflammation can lead to damage to the cerebrovascular endothelium, disrupting tight junction proteins and ultimately causing BBB breakdown (Barichello et al., 2021). Tight junctions (TJ) are sturdy physical barriers formed between endothelial cells and are an essential component in maintaining BBB integrity and normal function (Sasson et al., 2021). Infection indirectly leads to TJ degradation or disruption through various pathways. For example, TNF-α and IL-1β in peripheral inflammation can cause a reduction in TJ expression or incorrect TJ distribution (Presta et al., 2018). Claudin-5, an antibody responsible for selective permeability of the BBB, undergoes downregulation and BBB disruption due to peripheral inflammation (Nitta et al., 2003). Additionally, IL-1β can cause claudin-5 to migrate across endothelium (Labus et al., 2018). In addition to claudin-5, occludin and zonula occludens-1 (ZO-1) are also vital junction proteins in the BBB. Inflammatory cytokines IL-1β and IL-8 cause the loss of these tight junction proteins occludin and ZO-1, increasing BBB permeability (Kim et al., 2022). Studies using a PND mouse model have shown a significant decrease in claudin-5 and occludin in brain tissue, as well as an increase in NK cells and CD4+ cells in the hippocampus, confirming that peripheral inflammatory cells entering through a damaged BBB contribute to hippocampal neuronal injury or inflammation, aggravating PND (Zhu et al., 2018). Furthermore, in elderly mice, the axon guidance molecule netrin-1 weakens the increased BBB permeability caused by peripheral inflammation by upregulating the expression of tight junction-related proteins such as ZO-1, claudin-5, and occludin, ultimately improving postoperative cognitive impairment (Li et al., 2021).

In addition to the degradation and abnormal distribution of tight junction proteins, peripheral inflammation also affects endothelial cell function. For instance, the exosome-derived miR-1-3p from septic plasma induces endothelial dysfunction by targeting endoplasmic reticulum stress-related protein 1, promoting cell apoptosis and cytoskeleton contraction, and increasing monolayer endothelial permeability and membrane injury (Gao et al., 2021). Moreover, peripheral inflammation affects endothelial cell function through multiple signaling pathways. For instance, lipopolysaccharide (LPS) activates Toll-like receptor 4 (TLR4) in endothelial cells, triggering a series of interactions within signaling pathways, including the nicotinamide adenine dinucleotide phosphate oxidase/reactive oxygen species (ROS)/endothelial nitric oxide synthase pathway, ultimately leading to endothelial dysfunction (Grylls et al., 2021). Furthermore, peripheral inflammation can increase the expression of adhesion molecules on endothelial cells, such as Vascular Cell Adhesion Molecule-1, Intercellular Adhesion Molecule-1, and E-selectin. The upregulation of these adhesion molecules may increase the adhesion between peripheral inflammatory cells and endothelial cells, further compromising BBB integrity (Yousef et al., 2019). The breakdown of the BBB caused by peripheral inflammation allows inflammatory cytokines to penetrate the CNS, triggering neuroinflammation, neurotoxicity, and neuronal dysfunction. Additionally, peripheral inflammation may promote the transmission of tau proteins within the brain, causing neuropathological changes in specific brain regions and ultimately leading to memory impairment (Yu et al., 2021).

In conclusion, the breakdown of the BBB due to peripheral inflammation is a complex and multifaceted process that involves the interaction of various inflammatory mediators and signaling pathways. This breakdown allows inflammatory cytokines to enter brain tissues, triggering neuroinflammation and functional impairments that have detrimental effects on cognitive function.

### 4.2 Neural transmission pathways

In addition to crossing the BBB, in certain circumstances, inflammatory mediators can enter brain tissue through neural transmission pathways. This neural transmission pathway involves the vagus nerve and sympathetic nerve fibers.

Under peripheral inflammatory conditions, inflammatory mediators can be transmitted to CNS structures such as the brainstem and cerebral cortex by stimulating the afferent fibers of the vagus nerve (Rodrigues et al., 2014). This transmission pathway is commonly referred to as the vagus nerve-inflammatory reflex. The activity of the vagus nerve, through the release of neurotransmitters such as acetylcholine (ACH), can regulate the function of multiple brain regions, including the hypothalamus, pituitary gland, and amygdala (Song et al., 2019). By stimulating the vagus nerve, inflammatory mediators can alter the neural activity in these brain regions, thus influencing cognition and emotions (Gudernatsch et al., 2020). Additionally, the sympathetic nerve also plays a role in the neural transmission of inflammatory mediators. The afferent fibers of the sympathetic nerve can transmit inflammatory mediators to the brain, thereby affecting the function of the CNS. The activity of the sympathetic nerve is associated with the stress response and the stress hormone release. Peripheral inflammation can stimulate sympathetic nerve activity, leading to increased release of stress hormones such as adrenaline and noradrenaline, which in turn affect neuronal function (Reiche et al., 2004). When the cascading neural inflammatory response caused by peripheral inflammation is not properly regulated, sustained neural inflammation can interfere with synaptic plasticity, which is the foundation of learning and memory in cognition, resulting in PND (Saxena and Maze, 2018). Studies have found that cytokines, including TNF-α, IL-1β, IL-6, IL-17, prostaglandins, and macrophages, neutrophils, mast cells, etc., interact with sensory neurons, thereby altering the excitability, ion currents, and second messenger systems of these neurons (Schaible, 2014). In mice, intraperitoneal injection of LPS is widely used as a model of systemic inflammation, mimicking the natural response to infection (Lopes, 2016). One study showed that intraperitoneal injection of LPS in rats rapidly stimulates the transcription of IL-6 genes in the choroid plexus and periventricular organs, resulting in a neuroinflammatory response (Vallières and Rivest, 1997). It has been found that oral administration of *Escherichia coli* to mice leads to a significant increase in c-Fos expression in bilateral vagal ganglia neurons (Goehler et al., 2005). Another study showed that intraperitoneal injection of LPS in rats can stimulate the afferent signal of the vagus nerve, which can be traced back to the solitary nucleus and then reach other brainstem and forebrain regions (Marvel et al., 2004). Partial blockade of the neuroinflammatory response caused by acute peripheral inflammation was observed in mice after vagus nerve transection (Yang et al., 2022). MCs, as the “first responders” of the neural transmission pathway, are activated early in hippocampal neuroinflammation and BBB dysfunction induced by LPS (Wang et al., 2020; Yang et al., 2022). Studies have shown that conditioned medium from LPS-stimulated microglial cell line (P815) can induce primary astrocyte activation through the mitogen-activated protein kinase pathway signaling, resulting in the production of TNF-α and IL-6. Moreover, activated P815 cells can independently induce neuronal apoptosis and synaptic injury in astrocytes, leading to PND (Zhang et al., 2016).

### 4.3 Activation of the complement system

The complement system is a vital immune system that can be activated during infection, inflammation, and injury, triggering inflammatory and immune responses (Mellors et al., 2020). Activation of the complement system results in a range of biological effects, including chemotaxis, lysis, inflammatory reactions, and immune regulation. One major consequence of complement activation is the induction of inflammatory reactions, leading to immune dysfunction in the body (Ricklin et al., 2010). Additionally, complement activation can influence neurogenesis and regeneration, as well as CNS function (Rahpeymai et al., 2006; Stevens et al., 2007). The complement system plays a role in the occurrence and development of PND and is activated when peripheral inflammation occurs (Xiong et al., 2018). For example, CRP, a peripheral inflammatory marker, can be used to predict the risk of postoperative delirium and postoperative neurocognitive disorders (Ashraf-Ganjouei et al., 2020). CRP levels in plasma rise sharply when the body is infected, and it can activate and regulate the classical complement pathway (Sproston and Ashworth, 2018). Previous clinical studies have shown that bacterial, fungal, or viral infections can lead to early activation of complement component 3 (C3), resulting in depletion of plasma C3 and upregulation of C3 cleavage forms, including C3a and C3b (Conigliaro et al., 2019). Previous studies have shown that after intraperitoneal injection of LPS, levels of complement C3 in astrocytes and expression of C3a receptors in microglia in the hippocampus are specifically upregulated (Li et al., 2020). The C3/C3aR pathway has been implicated in various disease conditions, including virus-induced synaptic loss and tau pathology (Litvinchuk et al., 2018). Upregulation of hippocampal complement C3 is accompanied by a significant decrease in synaptic-related proteins and density (Ji et al., 2020). Upregulation of C3a receptors in microglia accelerates the deposition of amyloid-β in brain parenchyma, leading to synaptic dysfunction and cognitive decline (Pekna et al., 2021). Studies have shown that administration of a C3a receptor antagonist can improve hippocampus-dependent memory function, preserve the integrity of the BBB, and have a therapeutic effect on reducing neuroinflammation, suggesting the involvement of complement activation in the mechanism of PND occurrence (Xiong et al., 2018). In a mouse model of AD, C3 gene knockout reduced pro-inflammatory cytokines in the brain and synaptic loss near amyloid plaques, improving neurodegenerative pathologies (Shi et al., 2017). In C3-deficient mice induced with autoimmune encephalomyelitis, dendrites and spines in the dentate gyrus were preserved, along with memory capability (Bourel et al., 2021). Another study in a mouse model of stroke showed that administration of SB 290157 trifluoroacetic acid, a selective complement inhibitor, prevented microglia from engulfing stressed neurons and improved neuroinflammation and cognitive function, indicating the potential effectiveness of complement system inhibitors in preventing and treating cognitive impairments (Surugiu et al., 2019). Knockout of the C3 gene and specific inhibitors of complement components may become new therapeutic targets for improving PND.

### 4.4 Neuronal inflammatory response

Under peripheral inflammatory conditions, the continuous release of inflammatory mediators may result in sustained activation of the neuronal inflammatory response, causing direct damage to neurons (Vasunilashorn et al., 2021). Excessive release of inflammatory mediators can activate neuroglial cells, leading to increased release of more inflammatory mediators and oxidative stress response. For instance, inflammatory mediators such as IL-1β, TNF-α, and IL-6 can stimulate neuroglial cells to release a range of pro-inflammatory and toxic factors, including ROS, NO, and apoptotic factors, thereby leading to neuronal damage or death (De Biase et al., 2017; Liddelow and Barres, 2017; Bachiller et al., 2018).

Microglia, the crucial brain-specific cells, play a vital role in brain development by maintaining the neuronal microenvironment and participating in the phagocytic activity of neural precursors (Harry, 2013). Activated microglia in the aging brain can exhibit both neuroprotective and neurotoxic effects (Dilger and Johnson, 2008). In the process of neurodegeneration, the phagocytic function of microglia is beneficial in clearing debris and protein aggregates, but the clearance of live synapses and neurons is harmful (Butler et al., 2021). Activation of microglia is a hallmark of brain pathology (Dheen et al., 2007). The “protective-toxic” characteristics of activated microglia are associated with whether these cells are in a balanced or dysregulated state (Silvin and Ginhoux, 2018). Peripheral inflammation can lead to an imbalance in the phagocytic ability of microglia, and dysregulated microglia can excessively phagocytize neuronal dendritic spines (Cao et al., 2021). Intraperitoneal injection of LPS induces various central effects, primarily mediated by pro-inflammatory cytokines released from microglia (Catorce and Gevorkian, 2016). In a series of experiments, intraperitoneal injection of LPS was used to simulate a peripheral inflammatory environment, resulting in excessive expression of the pro-inflammatory cytokine IL-1β in the microglia of aged mice, ultimately leading to neuroinflammation (Henry et al., 2009). Another study indicated that peripheral inflammation triggered by LPS administration induces excessive activation of microglia, which in turn leads to the loss of dopaminergic neurons, inflammation, and neurodegeneration (Garcia-Dominguez et al., 2018). Peripheral inflammation also activates nicotinamide adenine dinucleotide phosphate oxidase in microglia, resulting in increased induction of mitochondrial ROS, further leading to neurotoxicity (Agrawal and Jha, 2020). In order to examine whether the inflammatory response of microglia induced by chronic peripheral inflammation is reversible, the team led by Patrick Süβ from the University of California, San Diego, injected an anti-TNF-α antibody, infliximab, targeting human TNF-α, into the peripheral tissues of Tg197 mice. They observed the loss of transcriptional signals in inflammatory microglia, indicating that the phenotype of microglia can be restored by inhibiting peripheral TNF-α (Schlachetzki et al., 2021).

Astrocytes, the most abundant cell type in the CNS, possess diverse functions, including neurotransmitter cycling, formation and maintenance of the BBB, immune signaling, and regulation of neuronal synaptogenesis (Giovannoni and Quintana, 2020). Studies have shown that under the influence of peripheral inflammation, astrocyte metabolism coupling is disrupted, leading to PND (Femenia et al., 2018). A common characteristic of reactive astrocytes is the upregulation of glial fibrillary acidic protein (GFAP) and CNS-specific protein (s100β), which are widely used as markers of CNS injury after trauma (Du Preez et al., 2021). The levels of GFAP and s100β are elevated in patients with PND compared to before surgery (Huang et al., 2021). Under the influence of peripheral inflammation, GFAP astrocytes in the radiatum of the hippocampus undergo morphological changes, presenting shorter processes and reduced GFAP coverage. These changes are completely reversible within 72 h postoperatively (Terrando et al., 2013). Moreover, under the influence of peripheral inflammation, microglia can activate A1 astrocytes through IL-1β, C1q, and TNF-α, thereby influencing synaptic and overall neuronal plasticity and leading to postoperative neuroinflammation (Xu et al., 2018). Furthermore, peripheral inflammation-induced activation of the C3/C3aR signaling in the CNS leads to the release of a large amount of C3 by astrocytes, which in turn promotes the activation of microglia, exacerbating postoperative neuroinflammation (Wei et al., 2021). Furthermore, inflammatory mediators can also exert various deleterious effects on neurons by activating receptors on the neuronal surface. For example, the inflammatory mediator TNF-α can increase intracellular calcium levels by activating TNF-α receptors on neurons, leading to neuronal damage or even death (Clark and Vissel, 2016; Batista et al., 2019).

### 4.5 Imbalance of neurotransmitters

In addition to the direct effects on neurons, the presence of inflammatory mediators in a state of peripheral inflammation can also disrupt the balance of neurotransmitters, thereby influencing cognition and emotions. The impact of the inflammatory mediator on the cholinergic system serves as a crucial link between peripheral inflammation and cognitive function. IL-1β can reduce the synthesis, release, and activity of acetylcholinesterase (ACHE), thus leading to a decrease in the levels of ACH (Gomes de Andrade et al., 2018). ACH plays a significant role in the cholinergic system, particularly in processes such as learning, memory, and cognition. Numerous experimental studies have demonstrated the association between a decline in ACH levels and a decrease in cognitive function. For instance, a considerable number of ACH neurons are present in brain regions closely associated with cognitive function, such as the cerebral cortex and hippocampus. The reduction of ACH may affect the normal function of these brain areas (Grantham and Geerts, 2002). Additionally, ACH is involved in the regulation of synaptic plasticity, playing a crucial role in learning and memory processes. Insufficient activity of ACH neurons can impair synaptic plasticity, thus affecting the formation and storage of memory (Schliebs and Arendt, 2011). The influence of the inflammatory mediator on the cholinergic system may be related to the onset mechanism of AD. AD is a chronic progressive neurological disorder characterized by a gradual decline in cognitive function and degenerative changes in neurons (Scheltens et al., 2016). Excessive release of TNF-α, IL-1β, IL-6, CXCL1, and HMGB1 may hasten the progression of AD, impairing the cholinergic system through a reduction in ACH levels and direct effects on cholinergic neurons (Bartus, 2000; Zaghloul et al., 2017). In fact, the cholinergic system, as a therapeutic target for AD, can be modulated by cholinesterase inhibitors such as ACHE and butyrylcholinesterase. These inhibitors can increase ACH levels and, to some extent, improve symptoms related to cognitive function (Di Santo et al., 2013).

In addition to impacting the cholinergic system, the presence of inflammatory mediators can also disrupt the balance of neurotransmitters glutamate (GLU) and gamma-aminobutyric acid (GABA), thereby influencing neuronal activity and cognitive function (Wohleb, 2016). GLU and GABA are the most common excitatory and inhibitory neurotransmitters within neurons, and maintaining a balanced relationship between them is crucial for neuronal activity. Inflammatory mediators such as TNF-α, IL-1β, and IL-6 can interfere with the balance of GLU and GABA through various mechanisms. Firstly, inflammatory mediators can regulate the expression and activity of GLU transporters and GABA transporters, thus affecting the distribution and concentration of GLU and GABA within neurons (Pitt et al., 2003; Tilleux and Hermans, 2007; Ida et al., 2008). For example, IL-1β increases intraneuronal GLU concentration by reducing the expression of GLU transporters, while the excessive release of TNF-α can decrease intraneuronal GABA concentration by altering the activity of GABA transporters (Das, 2003; Kołosowska et al., 2016). Secondly, inflammatory mediators can directly affect the receptors and ion channels of GLU and GABA, thereby altering neuronal excitability and inhibition. For instance, inflammatory mediators like IL-1β, IL-6, and TNF-α can influence the activity, quantity, and subtype selection of GLU receptors, thus changing the function of intraneuronal GLU (Wang et al., 2000; Vezzani et al., 2011). Similarly, inflammatory mediators can also affect the expression and activity of GABA receptors and ion channels, thereby influencing the effects of intraneuronal GABA (Wang et al., 2000).

### 4.6 Gut-brain axis

The gut-brain axis is a two-way communication system, which explains how through the vagus nerve, the gut microbiota can affect the CNS, including brain functions related to the enteric nervous system, as well as how CNS can alter various gut secretions and immune responses (Fleck et al., 2017). The gut microbiota is a key component of the gut-brain axis. The association between disturbed gut microbiota and PND is currently a hot topic in research (Dong et al., 2021). The gut microbiota is a collection of microorganisms, including bacteria, fungi, viruses, and others, living in the human gut and having a symbiotic relationship with the host, playing an important role in human health and disease development (Chen et al., 2021). Inflammatory gut microbiota in the periphery produces metabolites through different metabolic pathways, such as short-chain fatty acids, amino acids, and bioactive substances, these metabolites can directly or indirectly affect communication within the gut-brain axis, thus affecting the degree and development of cognitive function (Agus et al., 2021).

When the body suffers from infection, trauma, or other diseases, the immune system releases inflammatory mediators, such as IL and TNF, which can affect the composition and function of gut microbiota, even leading to increased gut permeability, allowing the gut microbiota and its metabolites to enter the bloodstream, further inducing systemic inflammatory reactions (Ji et al., 2023). Gut microbiota, through neurons, endocrine and immune systems, transmits information to the CNS, thus affecting the function of the entire nervous system. Gut microbiota can also regulate the activity of the immune system through metabolite regulation. The immune system is a crucial component of the body’s fight against inflammation and infection and also participates in regulating nervous inflammation. The gut microbiota stimulates the immune system to produce immune factors and inflammatory mediators, such as TNF-α and IL-1β, which are transported to the brain through the blood and nerve pathways, leading to neuroinflammatory responses (Emanuele et al., 2010; Brown, 2019). On the other hand, gut microbiota also influences the degree of nervous inflammation by regulating gut barrier function. The gut barrier is a protective barrier formed by the intestinal epithelium and mucosal layer, which can prevent harmful substances from penetrating (Goto and Kiyono, 2012). When the gut barrier function is damaged, bacteria and toxins can penetrate the intestinal wall and activate the immune system, leading to inflammation reactions, these inflammatory reactions can be transmitted to the brain through the gut-brain axis, causing nervous inflammation and cognitive dysfunction (Bajic et al., 2018; Jordan et al., 2018).

### 4.7 Abnormalities in vascular function and hemorheology

In a state of inflammation, peripheral blood vessels can become disrupted and the presence of inflammatory mediators can trigger inflammatory reactions and damage to the endothelial cells of the blood vessel wall, leading to changes in endothelial function and vascular contractility (Sørensen and Borregaard, 2016). These changes may result in abnormal increases or decreases in vascular tension, subsequently affecting cerebral microcirculation and blood supply (Gilberti et al., 2017). Endothelial cells, which comprise a layer of cells on the inner lining of blood vessels, possess critical physiological functions including maintenance of vascular wall integrity, regulation of vascular contractility and lumen diameter, among others (Michiels, 2003). However, in a state of inflammation, the release of inflammatory mediators such as cytokines and chemokines can stimulate endothelial cells and cause inflammatory reactions and damage (Prabhu and Frangogiannis, 2016). Research has shown that the presence of inflammatory mediators can lead to endothelial cell inflammation, characterized by cell proliferation, exudation and damage (Zhu et al., 2019). These changes not only damage the integrity of the vascular endothelial barrier, but also lead to changes in endothelial function such as increased vascular permeability and promotion of platelet and leukocyte adhesion, further exacerbating the development of peripheral inflammation (Ross, 1999). Moreover, the presence of inflammatory mediators can also lead to changes in vascular contractility. On one hand, the release of inflammatory mediators such as vasoactive substances (such as angiotensin and platelet activating factor) increases vascular constriction, causing abnormal increases in vascular tension (Hermant et al., 2003). On the other hand, the release of certain inflammatory mediators such as nitric oxide is inhibited, leading to a decrease in vascular relaxation function and reduced vascular tension (Neumann et al., 2004). The presence of inflammatory mediators can also increase plasma viscosity, red blood cell aggregation and platelet activation, leading to an increase in blood flow resistance and a decrease in microcirculatory perfusion, subsequently affecting the normal function of cerebral microcirculation and reducing the supply of oxygen and nutrients (Kotan et al., 2022; Wang et al., 2022). Ultimately, these changes in vascular function and abnormal vascular contractility can impact cerebral microcirculation and blood supply. The brain is a highly metabolic organ and is very sensitive to the supply of oxygen and nutrients. Therefore, vascular dysfunction under peripheral inflammatory conditions leads to phenomena such as microvascular spasm, increased permeability, and decreased blood flow, ultimately affecting cerebral microcirculation and blood supply. A lack of oxygen and nutrient supply to specific regions of the brain can lead to the inactivation and functional damage of neuron in that area, resulting in cognitive function disorders such as decreased memory, lack of concentration, and delayed thinking (Zhang et al., 2018).

## 5 General anesthetics and auxiliary drugs regulate peripheral inflammation

During the process of general anesthesia, drugs enter the patient’s circulatory system through inhalation or intravenous infusion, thereby affecting the body’s immune system and inflammatory response (Table 1). Some studies have found that certain general anesthesia drugs can regulate peripheral inflammatory responses by inhibiting the production and release of inflammatory mediators (Rossaint and Zarbock, 2018). Inhaled anesthetic sevoflurane has been studied and found to inhibit the production and release of cytokines such as TNF-α, IL-1β, and IL-6 (Schilling et al., 2011). This inhibitory effect may be achieved through the suppression of transcription factors likeNF-κB, which interferes with cytokine synthesis and release (Xu et al., 2016). Similarly, other inhaled anesthetics like isoflurane exhibit similar effects and have the ability to suppress peripheral inflammatory responses (Bedirli et al., 2018). They can regulate immune responses by reducing cytokines produced by inflammatory cells, and may exert their effects through activating the Nrf2/ARE pathway to alleviate endothelial cell oxidative stress and inhibit the expression of inflammation-related genes (Chen et al., 2006).

**Table 1:** General anesthetics and auxiliary drugs regulate peripheral inflammation.

Classification	General anesthesia drugs	Effects	Target points	References
Inhalation anesthetic	Sevoflurane	Inhibits the production and release of cytokines TNF-α, IL-1β, IL-6, etc.	NF-κB	Xu et al. (2016)
	Isoflurane	Inhibits the production and release of cytokines TNF-α, IL-1β, IL-6, etc.	Nrf2/ARE	Chen et al. (2006)
Intravenous anesthetic	Propofol	Inhibits the production and release of cytokines TNF-α, IL-1β, IL-6, IL-8, etc.	NF-κB; ROS; Nox2; endothelial cells	Hsing et al. (2011), Yao et al. (2018), and Fan et al. (2020)
Opioid analgesics	Fentanyl and sufentanil	Inhibits leukocyte chemotaxis, activation, and signal transduction	Immune cells	Grimm et al. (1998), Brack et al. (2004), and Wen et al. (2021)
α2-adrenergic agonist	Dexmedetomidine	Inhibits the production and release of inflammatory mediators and cytokines such as iNOS, NO, IL-1β, and TNF-α	M1 microglia	Huang et al. (2018) and Bao et al. (2019)
	Clonidine	Inhibits the release of norepinephrine, glutamate, substance P, and pro-inflammatory cytokines	Primary afferent fibers	Lavand’homme and Eisenach (2003)
	Tizanidine	Inhibits the production of pro-inflammatory cytokines	TLR4/NF-κB	Talakoub et al. (2016) and Pei et al. (2018)
Anti-TNF-α therapy drugs	TNF-α antibodies and soluble TNF receptors fusion protein	Reduces the production and release of inflammatory mediators	TNF-α	Marino et al. (2020)
IL-6R monoclonal antibody	Tocilizumab	Reduces the production and release of inflammatory mediators	Cytokines	Langley et al. (2014)
IL-17A monoclonal antibody	Secukinumab	Inhibits the infiltration of neutrophils into the brain	Neutrophils	Katayama (2020)

Propofol is a commonly used intravenous general anesthetic. Besides its anesthetic effects, it also plays a role in regulating peripheral inflammation. Research has shown that propofol can inhibit the production and release of certain cytokines, such as TNF-α, IL-1β, IL-6, and IL-8 (Jia et al., 2017). These cytokines are key components of the inflammatory response. The mechanism by which propofol inhibits cytokine production and release may involve several aspects. Firstly, NF-κB is an important transcription factor involved in the regulation of inflammatory response-related gene transcription, propofol can inhibit the activation of transcription factors like NF-κB, thereby reducing the expression of pro-inflammatory genes (Hsing et al., 2011). Secondly, propofol possesses antioxidant capabilities, which can reduce oxidative stress and further alleviate peripheral inflammation. Research has shown that propofol can serve as a ROS scavenger to reduce oxidative stress, and it can also inhibit Nox2 to reduce the production of ROS that occurs subsequently (Yao et al., 2018). Additionally, propofol can also protect endothelial cell function. Endothelial cells, located within blood vessels, play a crucial role in maintaining normal vascular function and preventing inflammatory responses (Figarola et al., 2014). Research indicates that propofol can reduce endothelial cell inflammatory reactions and damage, helping to preserve normal vascular function (Fan et al., 2020).

Opioid drugs such as fentanyl and sufentanil are commonly used analgesics. They exert their analgesic effects by binding to opioid receptors in the CNS, but they also have an impact on the immune system. Studies have found that opioid drugs have anti-inflammatory effects and can inhibit leukocyte chemotaxis, activation, and signal transduction, thus alleviating peripheral inflammation (Grimm et al., 1998). Specifically, opioid drugs can bind to opioid receptors on immune cells, inhibiting their activity and function (Wen et al., 2021). This can reduce the release of inflammatory mediators such as TNF-α, IL-1β, and IL-6, thereby reducing the intensity of the inflammatory response. Moreover, opioid drugs can diminish immune cell chemotaxis and infiltration, thus mitigating the inflammatory process (Brack et al., 2004). However, it is important to note that opioid drugs also possess immunosuppressive effects. Prolonged or excessive usage of cocaine can have an impact on the body’s immune response to infections, increasing the risk of infection, particularly with the Human Immunodeficiency Virus (Cabral, 2006). Additionally, opioids are known to inhibit the activity of NK cells and macrophages, leading to decreased production of antibodies, IL-2, and interferon gamma, thereby undermining the body’s immune defenses (Sacerdote, 2008).

Alpha-2 (α2) agonists are commonly used sedative drugs in clinical anesthesia, they produce analgesic and anesthetic effects by stimulating α2-adrenergic receptors in the CNS (Bhana et al., 2000). Research has found that in cultured microglia activated by LPS, the commonly used α2 agonist dexmedetomidine inhibited the production and release of inflammatory mediators and cytokines including inducible nitric oxide synthase or NO, IL-1β, and TNF-α in a dose-dependent manner, while impeding the M1 activation of microglial cells and enhances their phagocytic activity (Huang et al., 2018; Bao et al., 2019). Clonidine is another kind of α2 adrenergic agonist that acts on the nerve terminals of primary afferent fibers, it can inhibit the release of norepinephrine, GLU, substance P, and pro-inflammatory cytokines, thereby reducing peripheral inflammation (Lavand’homme and Eisenach, 2003). Tizanidine is a muscle relaxant with α2 agonist analgesic mechanism with anti-nociceptive effect in neuropathic pain through inhibition of pro-inflammatory cytokines production via suppression of TLR4/NF-κB activation (Talakoub et al., 2016; Pei et al., 2018). By inhibiting cytokine production and release, α2 agonists can alleviate inflammation, playing a significant role in anesthesia and postoperative pain management.

In addition to the regulatory effects of general anesthesia drugs themselves, certain drugs targeting inflammatory mediators are also used as adjuvants to modulate peripheral inflammatory response during general anesthesia. These drugs include Anti-TNF-α therapeutic agents and anti- IL antibodies, which disrupt the cytokine signaling pathway and reduce the impact of inflammatory mediators on cells and tissues. Anti-TNF-α therapeutic agents are drugs that can neutralize the activity of TNF-α, such as TNF-α antibodies and soluble TNF receptors (Tracey et al., 2008). TNF-α is an important cytokine in the inflammatory process, participating in immune cell activation and release of inflammatory mediators (Holbrook et al., 2019). The application of anti-TNF-α therapeutic agents can inhibit the biological activity of TNF-α, thus alleviating peripheral inflammation and tissue damage (Marino et al., 2020). Another class of commonly used drugs targeting inflammatory mediators is anti-IL antibodies. Tocilizumab, is a recombinant humanized Anti- IL-6R monoclonal antibody which has a main use in the treatment of rheumatoid arthritis, systemic juvenile idiopathic arthritis and polyarticular juvenile idiopathic arthritis (Sheppard et al., 2017). Anti-IL antibodies can bind to these cytokines, blocking their biological activity, thereby reducing the production and release of inflammatory mediators and decreasing the intensity of the inflammatory response (Langley et al., 2014). Secukinumab, an antibody medication targeting IL-17A, is used to treat certain immune-mediated conditions. This medication can interfere with the transport of neutrophils from the bone marrow to the blood, thereby inhibiting neutrophil infiltration into the AD brain (Katayama, 2020). Studies indicate that Secukinumab provides benefits in improving cognitive function and alleviating inflammatory responses in AD patients, but further clinical research is required to confirm its effectiveness (Reichert, 2013). The combined use of these drugs targeting inflammatory mediators with general anesthesia drugs can synergistically modulate peripheral inflammatory response, further mitigating the impact of inflammatory mediators, reducing tissue inflammation, and improving the efficacy of anesthesia and surgical success rate.

## 6 Some preventive strategies for the peripheral inflammation pathogenesis of perioperative neurocognitive disorder

Recent studies have suggested that PND following general anesthesia may be related to peripheral inflammatory responses. Therefore, interventions targeted at peripheral inflammation may help prevent the occurrence of PND. The following are some preventative measures based on peripheral inflammation and the progress of related research (Table 2).

**Table 2:** Preventive strategies from different perspectives for the peripheral inflammation mechanism of PND.

Research focus	Basis and principles	Specific strategies	References
Anti-inflammatory Drugs	COX-1 and COX-2 enzymes	Salicylic acid	Lim et al. (1999)
	AMPK alpha/NF-κB	Flurbiprofen	Huang et al. (2022)
	Neurotransmitters, Inflammatory response	Dexamethasone	Valentin et al. (2016), Glumac et al. (2021), and Macks et al. (2022)
	Inflammatory mediators	Hydrocortisone	Li et al. (2023)
	Neurotransmitters, Inflammatory response	Testosterone	Zheng (2009) and Morozova et al. (2022)
	Anti-inflammatory activity in inflammation response	Erythropoietin	Lee et al. (2017)
Antioxidants	Oxidative stress and oxygen free radicals	Antioxidants such as Vitamin C, Vitamin E, Dopamine	Matsumoto (2015), Farina et al. (2017), and Zhang et al. (2018)
Immunosuppressants	Anti-inflammatory activity in inflammation response	Cyclosporine A and Azathioprine	Dobrowolski et al. (2023) and Tapia-Monsalves et al. (2023)
Lifestyle and Nutritional Status	Oxidative stress and oxygen free radicals, Probiotics, Blood sugar and lipids	Diet pattern rich in vegetables, fruits, whole grains, healthy fats and proteins	Swanson et al. (2012), Duda-Chodak et al. (2015), Handing et al. (2015), and Mirmiran et al. (2018)
	Immune system, Gut ecology	Moderate aerobic exercise	Shojaei et al. (2011), Simpson et al. (2016), Scheffer and Latini (2020), Lai et al. (2021), Zhang et al. (2021)
	Inflammatory response, Immune system	Weight control	Beavers et al. (2015)
	Inflammatory response, Immune system	Good sleep	Benington (2000) and Krueger and Obal (2003)
Recent molecular and cellular perspectives	Immune cells, Oxidative stress, Cell apoptosis, NF-κB	SIRT1	Zhou et al. (2006), Kitada et al. (2016), Ren et al. (2019), Sun et al. (2022), and Ye et al. (2022)
	Anti-inflammatory activity in inflammation response	TLRs (TLR2 and TLR4)	Hoogland et al. (2015)
	Target genes in inflammatory response	miRNA (miR-127, miR-146a, miR-124, miR-381)	Chen et al. (2019a,b), Loppi et al. (2021), and Wang et al. (2021)
	Inflammatory mediators	Acetylcholine	Hu et al. (2021)
	Immune cells	Vitamin D	Gianforcaro and Hamadeh (2014) and Mousa et al. (2017)
	Anti-inflammatory activity in inflammation response	Nerve growth factor	Jurgens and Johnson (2012) and Minnone et al. (2017)

### 6.1 Drugs

Nonsteroidal anti-inflammatory drugs (NSAIDs) are a commonly used class of medications primarily employed for relieving inflammation and pain (Taketo, 1998). Factors such as general anesthesia and surgical trauma can activate the inflammatory response in patients, leading to the release of inflammatory mediators. These mediators may exert negative effects on the CNS, resulting in cognitive dysfunction. Recent studies have suggested that NSAIDs may play a beneficial role in improving PND. Cyclooxygenase (COX) enzymes are involved in the synthesis of inflammatory mediators such as prostaglandins, and their excessive production is associated with inflammation and related pathological processes, Sulindac, a NSAIDs exert their anti-inflammatory effects by inhibiting the activity of COX, including COX-1 and COX-2 (Lim et al., 1999). It has been demonstrated that Scholinic acid exhibits partial inhibition on the cognitive changes induced by LPS in both the water maze and passive avoidance tasks (Lee et al., 2008). In an experimental setting involving 18-month-old rodents, the administration of the NSAIDs sulindac has demonstrated its potential in diminishing neuroinflammation in the hippocampus and enhancing cognitive abilities (Mesches et al., 2004). Recent studies have elucidated that NSAIDs, such as flurbiprofen, possess the capacity to mitigate formalin-triggered inflammatory pain and mild cognitive dysfunction in rodents by exerting their influence on inflammatory agents and acidic-responsive ion channels through the signaling pathway of AMPK alpha/NF-κB (Huang et al., 2022). Furthermore, research suggests that the concomitant administration of antidepressants and non-steroidal anti-inflammatory drugs contributes to alleviating patients’ depressive and anxiety symptoms, as well as somatic manifestations. It also reduces inflammatory markers, enhances patients’ cognitive functions, and mitigates various neurodegenerative disorders (Dong et al., 2022).

Corticosteroids are a class of highly potent anti-inflammatory drugs commonly used to treat various inflammatory conditions. By affecting the cellular signaling pathways, they inhibit the synthesis of inflammatory mediators and suppress the activity of white blood cells, thus alleviating inflammation and related symptoms (Flammer and Rogatsky, 2011). Among these drugs, dexamethasone and hydrocortisone are the most commonly used. Dexamethasone is a synthetic glucocorticoid that exerts its effects through inhibition of inflammatory reactions and regulation of the immune system. Studies suggest that dexamethasone may ameliorate postoperative cognitive function impairment by modulating neurotransmitter release, attenuating neuroinflammatory response, and regulating synaptic plasticity (Valentin et al., 2016; Glumac et al., 2021; Macks et al., 2022). Similarly, hydrocortisone, also a synthetic glucocorticoid, is the biologically active form of dexamethasone. Some studies indicate that hydrocortisone can promote neurogenesis and neuronal survival while increasing synaptic plasticity, thereby enhancing cognitive abilities (Dinse et al., 2017). Additionally, Hydrocortisone can also modulate the release of neuropeptides and the speed of neuronal transmission, exerting positive effects on the processes of learning and memory (Flanigan et al., 2020). Research has also shown that hydrocortisone can reduce the levels of inflammatory mediators in the brain, alleviate neuroinflammation, and subsequently relieve memory impairment and depressive symptoms, an especially important consideration for elderly and other individuals affected by cognitive impairments (Li et al., 2023). Meanwhile, androgenic steroids are a class of hormones that exist in both male and female bodies, of which testosterone is the most typical. Studies reveal that testosterone can promote neurogenesis and neuronal survival, enhance synaptic plasticity and neuroprotection, and play a significant role in neurotransmitter regulation (Leranth et al., 2003). Elevated levels of testosterone can mitigate inflammatory responses and promote the release and action of neurotransmitters such as dopamine, norepinephrine, GLU, and GABA, thereby enhancing cognitive function (Zheng, 2009; Morozova et al., 2022). In the elderly population, decreased testosterone levels are often accompanied by loss of cognitive function (Lv et al., 2016). Studies show that sufficient levels of testosterone are necessary for normal cognitive functions, especially attention, executive function, and memory (Cappa et al., 1988; Moffat et al., 2002; Muller et al., 2005). Therefore, supplementing testosterone may be a beneficial strategy to improve cognitive impairment and deficits. These studies suggest that the application of corticosteroids can alleviate symptoms of cognitive decline, memory impairment, and reduced cognitive flexibility. However, specific treatment drugs and courses require further research for clarity. The use of corticosteroids should be carefully considered as they may cause a series of side effects, including adrenal suppression, elevated blood sugar, osteoporosis, and immune function inhibition (Bleecker et al., 2020). Moreover, long-term use may lead to more serious side effects such as immune suppression-related infections and adrenal insufficiency (Liu et al., 2013).

In addition to NSAIDs and corticosteroids, other anti-inflammatory drugs are also being studied for their effects on the prevention and treatment of cognitive dysfunction. For instance, research has shown that erythropoietin, a hematopoietic hormone, has anti-inflammatory and neuroprotective effects, and has been demonstrated to prevent PND (Lee et al., 2017). Due to the involvement of oxidative stress and the generation of ROS in the mechanisms underlying PND, antioxidants are considered to have potential therapeutic effects. Some studies indicate that the intake of antioxidant substances or the application of antioxidant medications, such as vitamin C, vitamin E, and dopamine, can alleviate PND (Matsumoto, 2015; Farina et al., 2017; Zhang et al., 2018). The use of antioxidants in this context is widely researched. The immune system may also play a significant role in PND. Therefore, immunomodulators are also receiving attention for their potential in the prevention and treatment of cognitive dysfunction. For example, some studies have found that the use of immunosuppressants like cyclosporine A and azathioprine can alleviate cognitive dysfunction (Dobrowolski et al., 2023; Tapia-Monsalves et al., 2023).

### 6.2 Enhancing one’s lifestyle and nutritional status

Healthy diet: Adopting a healthy diet pattern, such as one rich in vegetables, fruits, whole grains, healthy fats, and proteins, can provide the body with a diverse array of essential nutrients, which can regulate inflammatory responses and improve cognitive function. Vegetables and fruits are rich in vitamins, minerals, and natural antioxidants such as vitamins C and E, which can neutralize harmful substances produced by free radicals, alleviate inflammatory responses, and protect nerve cells from oxidative stress (Mirmiran et al., 2018). Whole grains are an important source of dietary fiber, which can promote gut health, regulate gut microbiota, enhance the growth of beneficial bacteria, and reduce the proliferation of harmful bacteria (Duda-Chodak et al., 2015). This can help combat inflammatory responses and improve brain function. In addition, dietary fiber can also help control weight and regulate blood sugar and lipids, indirectly reducing the risk of inflammation (Satija and Hu, 2012). A diet rich in healthy fats (such as olive oil, fish, and nuts) is associated with reducing inflammatory responses and enhancing immune function. Among them, omega-3 fatty acids found in fish are considered an essential component in fighting inflammation and improving cognitive function (Swanson et al., 2012). Protein is an essential nutrient in the body, composed of amino acids. Studies have shown that a diet with an adequate intake of protein can promote the function of immune cells and balance inflammatory markers, thus improving postoperative cognitive impairment (Handing et al., 2015).

Moderate exercise: Research has shown that moderate aerobic exercise can lower postoperative inflammatory markers, such as CRP, white blood cell count, and cytokines, etc. (Shojaei et al., 2011). Exercise has the ability to modulate the immune system’s response, alleviate intestinal dysbiosis and butyric acid increase, enhance the production of anti-inflammatory factors, while reducing the release of inflammatory mediators, in order to maintain the appropriate level of inflammatory response, thus improving postoperative neuroplasticity and cognitive function under general anesthesia (Scheffer and Latini, 2020; Lai et al., 2021). Moderate exercise can also promote the migration of immune cells, especially increasing the number of immune cells in the blood, making it easier for them to reach infected or inflamed areas (Zhang et al., 2021). This can help improve the immune system’s response to infections and pathogens. In addition, exercise can enhance the interaction between immune cells, including signal transduction, cytokine release, and cell cooperation (Simpson et al., 2016). Moderate exercise can enhance the memory function of immune cells and improve the accuracy and speed of immune responses (Wang et al., 2020). Performing moderate physical activities, such as walking, jogging, swimming, etc., during the postoperative recovery period is believed to help lower the level of inflammation after general anesthesia and provide protection for cognitive function (Guszkowska, 2004). The latest study has revealed that aerobic exercise combined with chlorogenic acid exerts neuroprotective effects and reverses cognitive decline in the AD model mice (APP/PS1) through the SIRT1/PGC-1α/PPARγ signaling pathway (Shi et al., 2023).

Weight control: Weight control can lower the level of inflammation and improve cognitive function, especially during the postoperative recovery period (Burns et al., 2023). The obese population often exhibits elevated levels of inflammatory markers, attributed to adipocytokines and inflammatory mediators secreted by adipocytes such as leptin, adiponectin, and resistin (Peluso and Palmery, 2016). This form of low-grade chronic inflammation can exacerbate the development and deterioration of numerous chronic diseases, including cardiovascular disease, diabetes, and neurological disorders. Research indicates that individuals with obesity have lower immune cell activity and weaker ability to respond to pathogens (Richard et al., 2017). Obesity can lead to aberrant immune system function, including diminished antibody production, inflammatory responses, and cell cytotoxicity (Vandanmagsar et al., 2011; Milner and Beck, 2012). Additionally, obesity and excess adipose tissue can induce an inflammatory response, resulting in the production of a plethora of inflammatory mediators such as TNF-α, IL-6, and CRP (van Kruijsdijk et al., 2009). These inflammatory mediators can alter cerebral blood flow and impact the structure and function of neurons (Papadopoulos et al., 2000). Studies have found an association between obesity and cognitive decline, AD, and other neurological disorders, possibly due to obesity-induced inflammatory responses and cerebrovascular pathological changes (Mazon et al., 2017). By controlling weight, chronic inflammation levels can be reduced, lessening the impact of weight and fat burden on the immune system (Beavers et al., 2015). This not only enhances the immune system’s ability to respond to and clear pathogens but also lowers systemic inflammation levels post-general anesthesia, thus protecting postoperative cognitive function. Weight management contributes to decreasing chronic inflammation levels, improving cerebral blood flow and neuronal health, enhancing cognitive function, and reducing the risk of postoperative cognitive decline (Li et al., 2022).

Good sleep: Research indicates that inadequate sleep and poor sleep quality can disrupt immune system function. The sympathetic nervous system releases norepinephrine and epinephrine, thereby upregulating the secretion of pro-inflammatory biomarkers such as IL-6, CRP, IL-1β, and TNF-α (Irwin, 2002; Irwin and Opp, 2017). The elevation of these inflammatory markers is related to the development of many chronic diseases and cognitive decline. Adequate sleep is essential for maintaining and improving cognitive function. Sleep deprivation and poor sleep quality can negatively affect brain function, including memory, learning, attention, and concentration (Foster and Wulff, 2005; Alhola and Polo-Kantola, 2007). Current research suggests that chronic sleep deprivation can lead to an increase in brain inflammation and may potentially enhance the risk of cognitive decline and neurodegenerative diseases by increasing the burden of β-amyloid protein (Mander et al., 2016). Optimal sleep is crucial for the rejuvenation, maintenance, and clearance of neuronal metabolic byproducts in the brain. During the sleep cycle, the brain efficiently eliminates metabolic waste and neurotransmitters, aiding in the restoration of normal functionality in neurons and brain circuits (Krueger and Obal, 2003). Additionally, sleep fosters communication and information processing between brain cells, thereby facilitating memory consolidation and learning (Benington, 2000). These effects collectively contribute to the alleviation of PND associated with general anesthesia.

## 7 Latest advances in research at the cellular and molecular level

### 7.1 SIRT1

Silencing regulatory protein 1 (SIRT1) is an antioxidant enzyme associated with lifespan extension and metabolic homeostasis, and recent studies have revealed its crucial role in maintaining neural homeostasis and treating diseases (Cai et al., 2016). SIRT1 exhibits regulatory effects in peripheral inflammation, suppressing the release of inflammatory mediators and reducing inflammation. In a study on mice, it was observed that the expression level of SIRT1 decreased following cardiac bypass surgery, accompanied by the release of numerous cytokines and the occurrence of PND (Shi et al., 2020). However, even in the presence of damage and inflammation, activation of SIRT1 can protect the brain from neural injury and cognitive decline (Sun et al., 2022). Another study demonstrated that the expression level of SIRT1 decreased after infection, but its activators can inhibit the release of inflammatory mediators, preventing neural injury and cognitive impairment caused by excessive inflammatory response (Chen et al., 2022). Research indicates that SIRT1 regulates inflammatory responses and the release of inflammatory mediators through multiple mechanisms. Firstly, SIRT1 can inhibit the expression of inflammatory-related genes such as cytokines and factors related to inflammatory signaling pathways (Kitada et al., 2016). In recent studies on vascular dementia treatment, ligustilide has been shown to activate the AMPK/SIRT1 signaling pathway, ameliorate pathological changes in the hippocampus cells of dementia rats, promote remyelination, suppress the expression of inflammatory factors, and alleviate cognitive impairment following cerebral hemorrhage (Peng et al., 2022). Secondly, SIRT1 can regulate the production and release of inflammatory mediators, for example, by inhibiting the activation of NF-κB, thereby reducing the synthesis and release of inflammatory mediators (Ren et al., 2019). Research has found that the SIRT1 activator resveratrol can decrease the release of pro-inflammatory cytokines in microglial cells, therefore protecting cells from inflammatory damage. Its mechanism may involve SIRT1 assisting in reducing pro-inflammatory cytokines by inhibiting NF-κB (Yanez et al., 2017). Additionally, SIRT1 can also modulate the activity of immune cells, such as polarizing macrophages and cell apoptosis, further influencing peripheral inflammation (Ye et al., 2022). In a mammalian experimental study of Parkinson’s disease, the activator of Sirt1, resveratrol, can increase the expression of LC3 II, and plays a critical role in neuroprotection by enhancing autophagy to clear incorrectly folded proteins and impaired mitochondria within cells (Tang, 2016). Moreover, SIRT1 is closely linked to pathways associated with oxidative stress and cell apoptosis. Research has revealed that SIRT1 can enhance the expression of antioxidant enzymes, reduce the occurrence of oxidative stress, and alleviate peripheral inflammation by regulating pathways associated with cell apoptosis, such as the B-cell lymphoma-2 family and cytochrome C signaling, thereby safeguarding neurons from damage (Zhou et al., 2006). Liraglutide is a novel oral hypoglycemic agent, as research has indicated its ability to enhance the expression of AMPK and SIRT1 within cells. This, in turn, leads to the restoration of elevated levels of thiol reactive substance and reduced glutathione in the brain tissues of demyelinated mice (Elbaz et al., 2018). Mannose polysaccharide, derived from mangosteen, serves as an oxygen-heterocyclic ketone that can antagonize the accumulation of reactive oxygen species induced by glutamate by activating the AMPK/SIRT1/peroxisome proliferator-activated receptor gamma coactivator 1-alpha (PGC 1α) pathway (Zhu et al., 2019). PGC 1α, another crucial molecule associated with mitochondrial metabolism, can participate downstream of SIRT1 in the regulation of fundamental biological activities to protect nerve cells. Activation of the AMPK/SIRT1/PGC 1α pathway can increase the production of uncoupling protein 2, decrease ROS production, thereby reducing neuronal apoptosis caused by oxidative stress, leading to improvements in short-term behavioral deficits and long-term neurological function (Huang et al., 2019).

### 7.2 Toll-like receptors

TLRs are a class of receptors that play a crucial role in the immune system. TLRs are involved in signaling pathways during the process of inflammation, and their activity can lead to the release of inflammatory mediators and the generation of neuronal inflammation, ultimately resulting in PND (Lin et al., 2021). TLR4 is currently one of the most extensively studied immune inflammatory pattern recognition receptors, composed of an extracellular N-terminal domain rich in leucine residues, a transmembrane region, and an intracellular C-terminal domain. The extracellular N-terminal domain is capable of recognizing pathogen-associated molecular patterns such as lipopolysaccharides, viral RNA, as well as damage-associated molecular patterns like heat shock proteins and calpain (Takeuchi and Akira, 2010; Zhang et al., 2022). Research conducted by Lu et al. indicates that C57BL/6 mice exhibit cognitive deficits 48 h after tibial fracture, accompanied by an increase in hippocampal S100A8 expression. Furthermore, postoperatively, S100A8 and TLR4 co-localize in the spleen. On one hand, S100A8 can induce TLR4 activation, trigger TLR4/MyD88 signaling, and promote neuroinflammation and the occurrence of PND. On the other hand, the absence of TLR4 mitigates S100A8 protein-induced proliferation of hippocampal microglia, which is beneficial for the restoration of neuronal structure and function (Lu et al., 2015; Fei et al., 2022). Other studies have indicated that older mice exhibit impaired intestinal barrier function, leading to increased levels of TLR2 and TLR4 ligands in the portal and peripheral blood as well as in the hippocampus and prefrontal cortex, which subsequently results in cognitive impairment in the mice (Brandt et al., 2023). In addition to participating in the inflammation signaling pathways associated with the occurrence and development of PND, TLRs may also synergistically contribute to the onset of PND in other aspects. For instance, when peripheral inflammation stimulation (activated by TLR-2 and TLR-4) leads to the activation of microglial cells, they may play a modulatory role in cognitive function (Hoogland et al., 2015). Research by Rodríguez-Gómez et al. has shown that TLR4 can mediate the interconversion of two phenotypes of activated microglial cells, the classical activation (M1) and alternative activation (M2) (Rodríguez-Gómez et al., 2020). Following M1 activation, interleukin-1β and interleukin-6, among other pro-inflammatory factors, are secreted, exacerbating neuronal cell damage; M2, on the other hand, secretes anti-inflammatory factors such as interleukin-4 and TGF-β, clearing oxidative stress and improving the microenvironment for neuronal cell survival, thereby promoting neuroregeneration and repair (Orihuela et al., 2016). Furthermore, TLRs also play an important role in neurodevelopment and regeneration and exert inhibitory effects in the process of neuronal apoptosis (Liu et al., 2013; Liang et al., 2020). Research by Rolls and colleagues suggests that neural stem cells express TLR2 and TLR4 on their surface, and the changes in receptors determine the proliferation and differentiation direction of NSCs. When bromodeoxyuridine nucleoside was injected into the peritoneal cavity of TLR4 deficient mice (TLR4D) and control mice, it was observed after 7 days that the number of BrdU+ cells in the SGZ of TLR4D mice significantly increased, though there was no significant difference in the number of BrdU+ cells that differentiated into astrocytes and oligodendrocytes, indicating that the absence of TLR4 can significantly increase the proliferation of neural precursors into neurons, without affecting the differentiation process into mature neurons. Upregulation of TLR2 receptors or downregulation of TLR4 receptors is beneficial for NSCs to differentiate into neurons, and the absence or downregulation of TLR4 can significantly promote NSC proliferation (Rolls et al., 2007; Takeuchi and Akira, 2010).

### 7.3 MicroRNA

MicroRNAs (miRNAs) are a class of short non-coding RNA molecules that regulate gene expression (Karp and Ambros, 2005). As important molecules involved in gene expression regulation, miRNAs can modulate gene expression at the transcription or translation level by targeting specific mRNA (Zhang, 2015). MiR-127, an important inflammation-associated miRNA, is closely associated with inflammatory reactions and immune regulation (Ren et al., 2021). Notably, circulating miR-127-3p has been suggested as a biomarker for temporal lobe dementia (Piscopo et al., 2018). Studies have revealed that reduced levels of miR-127 in an animal model of peripheral inflammation-induced aging can compromise neuronal survival (Loppi et al., 2021). To evaluate the pathways targeted by miR-127, pathway analysis was performed on predicted targets of the miR-127-5p arm in both human and mouse arms, revealing proteasome Psmd3 as a target of miR-127-5p (Loppi et al., 2021). Notably, proteasome activity not only declines with normal aging but also decreases during periods of inflammation, according to the findings of this study (Stanimirovic et al., 2001).

Furthermore, other miRNAs such as miR-146a, miR217, miR-124, and miR-381 have been found to play a crucial role in inflammation-related cognitive dysfunction. They can regulate the expression of target genes related to inflammatory response, neuronal function, and synaptic plasticity, thereby influencing the occurrence and development of PND (Chen et al., 2019a,b; Wang et al., 2021). Research by Yuanyuan Liang and others has shown that increased expression of miR-146a may affect the encoding genes of leukocyte receptor-associated kinases and tumor necrosis factor receptor-associated factor 6 through the TLR4 signaling pathway, leading to increased expression of inflammatory factors TNF-α, IL-1β, and IL-6 in the hippocampal region (Liang and Wang, 2021). Another study has found that miR217 can reduce the expression of pro-inflammatory factors TNF-α, IL6, and IL10 through the TLR4/PI3K/Akt/NF-κB signaling pathway, thereby inhibiting inflammatory responses (Zhang et al., 2020). Furthermore, in an article published in Cell Research, SUN and colleagues discovered that nicotine can induce upregulation of miR-124 through activation of a7nAChR, subsequently inhibiting STAT3 tyrosine phosphorylation and protein expression, as well as reducing LPS-induced production of IL6 and TNF (Sun et al., 2013). Additionally, research reports that dexmedetomidine improves PND through the miR-381/EGFR1/p53 axis and protects against perioperative neurocognitive disorder induced by sevoflurane through the miR-129/TLR4 axis (Wang et al., 2021; Wei et al., 2021).

### 7.4 Cholinergic inflammatory pathway

ACH, an important neurotransmitter, has a close relationship with the inflammatory process. It can reduce the release of inflammatory mediators and the degree of inflammatory reactions by binding to cholinergic receptors (Yang et al., 2022). Recently, the cholinergic anti-inflammatory pathway (CAP) has received increasing attention in the prevention and treatment of neuroinflammation and cognitive decline caused by peripheral inflammation. It has been demonstrated through research that there is a correlation between excessive and persistent cognitive decline and inflammatory responses in aged mice, which is associated with impairments in CAP function (Gong et al., 2020). However, these phenomena can be reversed by the administration of α7nAchR agonists (Wang et al., 2021). A study reported that the development of inflammation in sevoflurane-induced cognitive decline is associated with the downregulation of alpha 7 nicotinic ACH receptor CAP in aged rats (Yin et al., 2019). Further research has indicated that inhaling sevoflurane can impair cognitive function in rats. Stimulation of the vagus nerve is known to activate the cholinergic system in the basal forebrain, thereby alleviating sevoflurane-induced apoptosis, necrosis, and microglial cell activation in the hippocampus (Zhou et al., 2023). In the CAP, cholinergic receptors play an important role, which include cholinergic M1, M2, M3, and M4 receptors. Activating cholinergic receptors can inhibit the release of inflammatory mediators and the production of neuroinflammatory responses. Studies have shown that activation of cholinergic receptors plays an important role in inflammation inhibition and protection of neurons by regulating the production of inflammatory mediators, suppressing neuronal inflammatory responses, and improving synaptic plasticity (Hu et al., 2021). In addition, ACHE is a key enzyme for the degradation of ACH, and its activity level is closely related to the CAP. The decreased activity of ACHE, as a biomarker of parasympathetic dysfunction and inflammation-related disorders, leads to the accumulation of ACH in the synaptic cleft, enhancing the effectiveness of the CAP (Pohanka, 2014; Shenhar-Tsarfaty et al., 2014). Donepezil is a cholinesterase inhibitor, which works by inhibiting acetylcholinesterase in the synaptic cleft, thereby reducing the hydrolysis of acetylcholine released from the presynaptic neuron into the synaptic cleft. This enhances stimulation of cholinergic receptors and is used to improve mild to moderate cognitive impairment (Schneider, 2000; Zhang and Gordon, 2018). Memantine, an NMDA receptor antagonist, modulates glutamate activity, and is used in the treatment of cognitive impairment in the middle to late stages (Uddin et al., 2021).

### 7.5 Vitamin D

Calcium and vitamin D (VD) are essential nutrients for regulating bone health, but recent studies have shown that VD is also involved in modulating neuroimmune system function (Landel et al., 2016). Research has found that VD plays a role in immune responses by regulating T cell activity, macrophage function, and leukocyte migration (Mora et al., 2008). Moreover, VD has anti-inflammatory effects and participates in regulating the occurrence and development of inflammation responses. VD, through its receptor VDR, regulates the function of immune cells and the expression of inflammatory mediators in the immune system (Mousa et al., 2017). Activation of VD can inhibit the production and release of inflammatory mediators, such as cytokines TNF-α and IL-6 (Calton et al., 2015). These cytokines play important regulatory roles in inflammation responses, but excessive inflammation response may lead to tissue damage and disease development. The research has shown that VD can inhibit the synthesis and release of peripheral and central TNF-α, thereby modulating the inflammatory response mediated by the innate immune system (Almeida Moreira Leal et al., 2020). VD also exhibits inhibitory effects on immune cells such as macrophages and lymphocytes, helping to alleviate peripheral inflammation and hinder the development of inflammation-related cognitive impairments (Yu and Cantorna, 2008). Furthermore, in the immune system, VD can regulate processes such as immune cell differentiation, proliferation, and migration, which have important influences on immune responses and immune tolerance. Immune cells express VDR, and through binding with VD, they affect gene expression and signaling pathways, thereby regulating the function of immune cells (Di Rosa et al., 2011). By modulating immune cell activity and inhibiting inflammation response, VD can alleviate the negative impact of peripheral inflammation on the brain and have positive effects on cognitive function (Gianforcaro and Hamadeh, 2014). Research indicates that the improvement of PND with Vitamin D is largely attributed to the inhibition of inflammatory CD4 T-cell lineages, T helper 17 cells, accompanied by the expansion of regulatory T cells, which are a vital subset of CD4 T cells in suppressing inflammation (Tian et al., 2015).

### 7.6 Nerve growth factors

Nerve growth factor (NGF) plays a crucial role in the development, regeneration, and formation of memory in the nervous system. It promotes the survival and growth of neurons by interacting with receptors, and is involved in regulating synaptic connections, neuronal development, and function (Allen et al., 2013). Peripheral inflammatory mediators can regulate neuronal function by affecting the synthesis and release of NGF. Studies have shown that cytokines and mediators produced during systemic inflammatory reactions may directly or indirectly lead to a decrease in NGF levels, thereby negatively impacting cognitive function (Elahi et al., 2020). In a mouse animal model, peripheral inflammation affects hippocampal synaptic plasticity, and NGF was found to reverse changes in synaptic plasticity caused by peripheral inflammation and improve cognitive function (Jurgens and Johnson, 2012). Additionally, NGF exerts anti-inflammatory effects through interactions with sensory neurons and is involved in the regulation and repair processes of inflammatory reactions (Minnone et al., 2017). NGF acts by activating the TrkA receptor to facilitate learning and memory (Kaplan and Miller, 2004). The TrkA protein is a crucial receptor tyrosine kinase, guiding the development and maturation of the nervous system, influencing neuronal proliferation, differentiation, and survival (Vaishnavi et al., 2015). Abnormal levels of TrkA have been reported in several neurodegenerative diseases, such as Alzheimer’s disease (Counts et al., 2004). Studies have indicated that the combined application of NGF and TRKA can activate the PI3K/MAPK/PLCγ signaling pathway, inhibit apoptosis proteins, promote neuronal differentiation and axon growth, and mediate allergic reactions (Nakagawara, 2001). Another study conducted in APP/PS1 mice found that activation of the NGF-TrkA pathway induces neurogenesis, regulates the c-Raf/ERK1-2/CREB cascade response, reduces Abeta levels, and improves cognitive impairment (Liu et al., 2014). Research has also shown that co-transfection of nerve growth factor and TERT in bone marrow mesenchymal stem cells promotes recovery of cognitive impairment in vascular dementia rats (Wang et al., 2014). Increasing the intake of neurotrophic factors can elevate the levels of neurotrophic factors in the CNS, thereby helping to protect cognitive function. For example, consuming fruits and vegetables rich in various vitamins, as well as fish rich in OMEGA-3 fatty acids, can increase the levels of NGF (Fukuoka et al., 2001; Huang et al., 2018). Furthermore, interventions with antidepressant drugs such as Olanzapine and Risperidone may also elevate the levels of neurotrophic factors, thus safeguarding cognitive function (Parikh et al., 2004).

## 8 Conclusion and future directions

The correlation and mechanism between PND and peripheral inflammation after general anesthesia is an important area of research. Current studies suggest that peripheral inflammatory responses may play a role in PND. On one hand, peripheral inflammation can impact the CNS through pathways such as neuroinflammation, signaling of inflammatory mediators, the complement system, and the gut-brain axis, thus exerting negative effects on cognitive function. On the other hand, during the process of general anesthesia, the selection and use of anesthetic drugs can to some extent decrease the release of inflammatory mediators and reduce the degree of inflammatory response. Therefore, in clinical practice, regulating peripheral inflammatory response and taking preventive measures are crucial. The use of anti-inflammatory drugs such as NSAIDs, corticosteroids, and vitamin D may help alleviate PND after general anesthesia. However, specific drug selection and treatment plans still require further research and validation. In addition to pharmacological interventions, improving patients’ lifestyle and nutritional status postoperatively is also crucial. This includes a balanced diet, moderate exercise, and good sleep. These measures can help reduce the risk of PND after general anesthesia. Many experiments are currently limited by the use of animal models, leading to uncertainties regarding the relevance, safety, and effective dosage information of treatments to human outcomes. Further research is still necessary to better understand the relationship between peripheral inflammation and PND after general anesthesia, and to establish the optimal strategies for inflammation regulation and prevention. Clinical trials and large-scale studies are needed to evaluate the safety and effectiveness of different prevention and treatment strategies. Organoid technology, as an emerging field, allows for the simulation of the three-dimensional structure and physiological function of organs or tissues *in vitro*, either in a normal or diseased state. This opens up possibilities for researchers to create disease models *in vitro*, with the potential application of this technology in studying the effects of peripheral inflammation on PND. Discrepancies in the measurement of inflammation currently exist within research, but future studies could establish unity by defining, assessing tools, and methods related to inflammatory markers associated with cognitive impairment, such as cytokines, inflammatory mediators, and inflammation-related genes. This unity would facilitate early diagnosis, monitor inflammatory status, and evaluate treatment efficacy. This will help guide clinical practice and improve the prognosis and quality of life for vulnerable patients.

## Author contributions

YL: Writing – original draft, Conceptualization, Visualization. Y-JL: Validation, Visualization, Writing – original draft. XF: Formal analysis, Writing – original draft. D-QC: Data curation, Writing – original draft. W-QY: Investigation, Writing – original draft. Z-QZ: Conceptualization, Supervision, Writing – review & editing.
